# A review and guide to creating patient specific 3D printed anatomical models from MRI for benign gynecologic surgery

**DOI:** 10.1186/s41205-021-00107-7

**Published:** 2021-07-05

**Authors:** Teresa E. Flaxman, Carly M. Cooke, Olivier X. Miguel, Adnan M. Sheikh, Sukhbir S. Singh

**Affiliations:** 1 Department of Clinical Epidemiology, Ottawa Hospital Research Institute, 1967 Riverside Dr, 7th Floor, Ottawa, ON K1H7W9 Canada; 2 Department of Obstetrics and Gynecology, Faculty of Medicine, University of Ottawa, Ottawa, ON Canada; 3 Department of Medical Imaging, The Ottawa Hospital, Ottawa, ON Canada; 4 Department of Radiology, Faculty of Medicine, University of Ottawa, Ottawa, ON Canada; 5 Department of Obstetrics, Gynecology and Newborn Care, The Ottawa Hospital, Ottawa, ON Canada

**Keywords:** MRI, Gynecology, Myomectomy, Endometriosis, 3D printing, Surgical planning, Barriers of use

## Abstract

**Background:**

Patient specific three-dimensional (3D) models can be derived from two-dimensional medical images, such as magnetic resonance (MR) images. 3D models have been shown to improve anatomical comprehension by providing more accurate assessments of anatomical volumes and better perspectives of structural orientations relative to adjacent structures. The clinical benefit of using patient specific 3D printed models have been highlighted in the fields of orthopaedics, cardiothoracics, and neurosurgery for the purpose of pre-surgical planning. However, reports on the clinical use of 3D printed models in the field of gynecology are limited.

**Main text:**

This article aims to provide a brief overview of the principles of 3D printing and the steps required to derive patient-specific, anatomically accurate 3D printed models of gynecologic anatomy from MR images. Examples of 3D printed models for uterine fibroids and endometriosis are presented as well as a discussion on the barriers to clinical uptake and the future directions for 3D printing in the field of gynecological surgery.

**Conclusion:**

Successful gynecologic surgery requires a thorough understanding of the patient’s anatomy and burden of disease. Future use of patient specific 3D printed models is encouraged so the clinical benefit can be better understood and evidence to support their use in standard of care can be provided.

## Background

Magnetic resonance imaging (MRI) and ultrasound (US) are the first lines of investigation for the diagnosis and monitoring of pelvic pathologies [1]. MRI and US are also used for pre-surgical planning of complex cases because they help a surgeon to visualize the patient’s anatomy, and location and extent of the disease [1]. Using medical imaging for pre-surgical planning of complex cases has been shown to reduce surgical complications and improve patient outcomes [2, 3]. However, for surgeons not experienced in reading 2D medical images, such as MRI and US, it can be difficult to appreciate complex anatomical structures and pathology in a way that can be applied to surgery [4].

Recent technological advancements in imaging have made it possible to generate three-dimensional (3D) constructs from two-dimensional (2D) images. By using specialized software, 2D medical images such as MRI, can be converted to 3D digital models. Rendering of 2D images to 3D digital models has been shown to significantly improve anatomical comprehension by providing more accurate assessments of anatomical volumes [5, 6], better perspective of structural orientations relative to adjacent structures [7], and improved visualization as transparency and colors can be modified to suit the user’s needs.

Furthermore, these 3D digital models can be converted into tangible, physical models by way of 3D printing [8, 9]. Rather than 3D visualization where a volumetric model is viewed on a 2D computer screen, a 3D printed model can provide a real indication of depth and tactile feedback, thus allowing surgeons to develop a clearer understanding of surgical anatomy [10, 11]. With a better visualization of disease location relative to adjacent organs, surgeons utilising 3D printed models for pre-operative planning have been shown to have greater surgical outcomes including, decreased operative time [12–14], blood loss [12, 13], and incision length [13].

The clinical benefits of using patient-specific 3D printed models for pre-surgical planning has been highlighted in the fields of orthopaedics [15], cardiothoracics [16, 17], and neurosurgery [18]. However, reports on the clinical use of 3D models in the field of gynecology are limited [19]. In this report, we will provide an overview of the principles of 3D printing and the steps required to derive patient-specific, anatomically accurate 3D printed models from MRI. We will then describe the application of 3D printed models for pre-operative planning for two benign gynecologic conditions: uterine fibroids and endometriosis, and discuss barriers to clinical update and future directions for 3D printing in gynecology.

## 3D Printing

### Basic principles

Three-dimensional (3D) printing, also known as additive manufacturing, is a process used to create a 3D object by adding material in a layer-by-layer process. This technology can be used to rapidly manufacture objects with complex shapes, at a fraction of the cost compared to traditional manufacturing methods [20]. Due to the reduced manufacturing costs of 3D printers and improved printing precision and speed, the industry has recently exploded, allowing for major advances in many industries including the medical field [21]. Popular medical applications include hearing aids, prosthetic limbs, surgical guides and implants, and detailed models of organs, bones, and blood cells, which can be printed in a variety of materials including polymers, metal, and ceramics, depending on the application [22, 23].

Several technologies have been established to create 3D printed products. These include vat photopolymerization, directed energy deposition, binder and material jetting, powder bed fusion, sheet lamination, and material extrusion (MEX), whereby MEX is the most common technique. The main considerations when choosing the type of 3D printing to use depends on the application of manufactured parts, machine cost, speed of printing, multi-material capabilities (or single material), and types of materials available.

To prepare a product for 3D printing a 3D digital object is needed because when printing, each layer contains a cross-section of the 3D object which are computed from the digital representation. To create a 3D digital object, a computer-aided design (CAD) software is required. Like the different printers and materials available, there are several options including licenced software (e.g. Materialise Mimics) and open-source applications (e.g. MITK Workbench, 3D Slicer), which depends on the users needs and functionality.

### General workflow for applications in medical radiology

In order to produce a patient specific and anatomically accurate 3D printed model, a high-resolution volumetric dataset is first required. Such datasets are typically stored as Digital Imaging and Communications in Medicine (DICOM) files. These files contain information about the medical image such as modality (type of imaging), contrast/gray values (in the form of 2D or 3D matrices), spacing between slices, spatial resolution, and anatomical orientation [24]. Volumetric DICOM data can be acquired from MRI, US, or computed tomography (CT). From this, a labelling process called segmentation is used to identify and isolate anatomy of interest. Segmentation can be done automatically using algorithms such as thresholding, edge detection, and region growing, or manually by essentially tracing anatomy of interest on each image slice. Most often, segmentation requires a semi-automatic approach whereby a combination of algorithms are used and manually verified [16]. Unfortunately, a reliance on manual or semi-automatic segmentation requires expertise and a significant time commitment on the part of the user.

The labels of the segmented DICOM images are then converted to 3D digital models using specialized software and then are saved in a format used for the 3D printing process such as stereolithography (STL), OBJect (OBJ), additive manufacturing file (AMF), or 3D manufacturing format (3MF). STL is the most commonly used format but cannot store color, material, or texture information and requires additional software to do so. The file type will depend on the software and printers being used to create the model. In general, 3D printing file types describe the surface geometry of a 3D object which contains information such as spatial position, scale, and orientation. You can have more than one 3D object within a 3D printing file, which is useful when including multiple anatomical structures in a model. These files are then recognized by the 3D printer’s supporting software. Here, the user can specify if 3D objects are to be printed individually or together, what colours the objects are to be printed as (if the file type and printer allows), and optimize the printing parameters to minimize material use and printing time. Printing times can vary depending on printer type, resolution, number of colours and materials used. After printing is complete, any support structure that was required to stabilize the model during the print is removed.

### 3D printing from MRI in gynecology

Our institution’s approach for deriving patient specific 3D printed anatomical models for applications in benign gynecology is described here. De-identified cross-sectional images from MRI post gadolinium (with additional 2 mm T2 weighted isometric sequenced images acquired for 3D printing purposes) are exported in DICOM format. DICOM files are imported into Materialise Mimics 20.0 (Materialise, Leuven, Belgium) software where relevant gynecologic structures and patient anatomy are segmented (i.e. separated) semi-automatically under the supervision of our teams’ fellowship-trained radiologists. We use a combination of signal thresholding, region growing and mask splitting algorithms to create basic masks (i.e. define overlapping objects) of anatomy of interest. Manual slice edits with interpolation are then conducted with direct planimetry to ensure accuracy of masks.

Once segmentation is complete, the 2D images are then converted to 3D objects to create a 3D digital model. Since we are doing multi-organ segmentation, we are dealing with multiple 3D objects with each 3D object being a different anatomical structure of interest. Each 3D object is exported to a separate STL file and then prepared for 3D printing via a computer-aided design (CAD) software 3-Matic (Materialise, Leuven, Belgium). The CAD process involves operations such as wrapping, smoothing and boolean operations (i.e. union, subtraction and intersection). The boolean subtraction operation is used to remove intersecting geometry of multiple 3D objects. For example, the endometrium model is subtracted from the uterus creating a cavity (i.e. negative space) in the uterus model. This is important to prevent unwanted overlapped material deposition during multi-material 3D printing.

The final digital model is then exported as multiple STL files and imported as an assembly into GrabCAD Print software (Stratasys, Inc., Eden Prarie, MN) for printing using a material jetting printer called the Connex 3 Objet 500 3D printer (Stratasys, Inc., Eden Prairie, MN). Our models are printed using a combination of transparent and opaque material (Vero line, Stratasys, Inc.) based on the gynecologist’s preference. For example, pathologic lesions or tissue of interest are printed in a bright opaque coloured material to maximize visualization in comparison to non-diseased anatomy printed in translucent clear material.

**Fig. 1:**
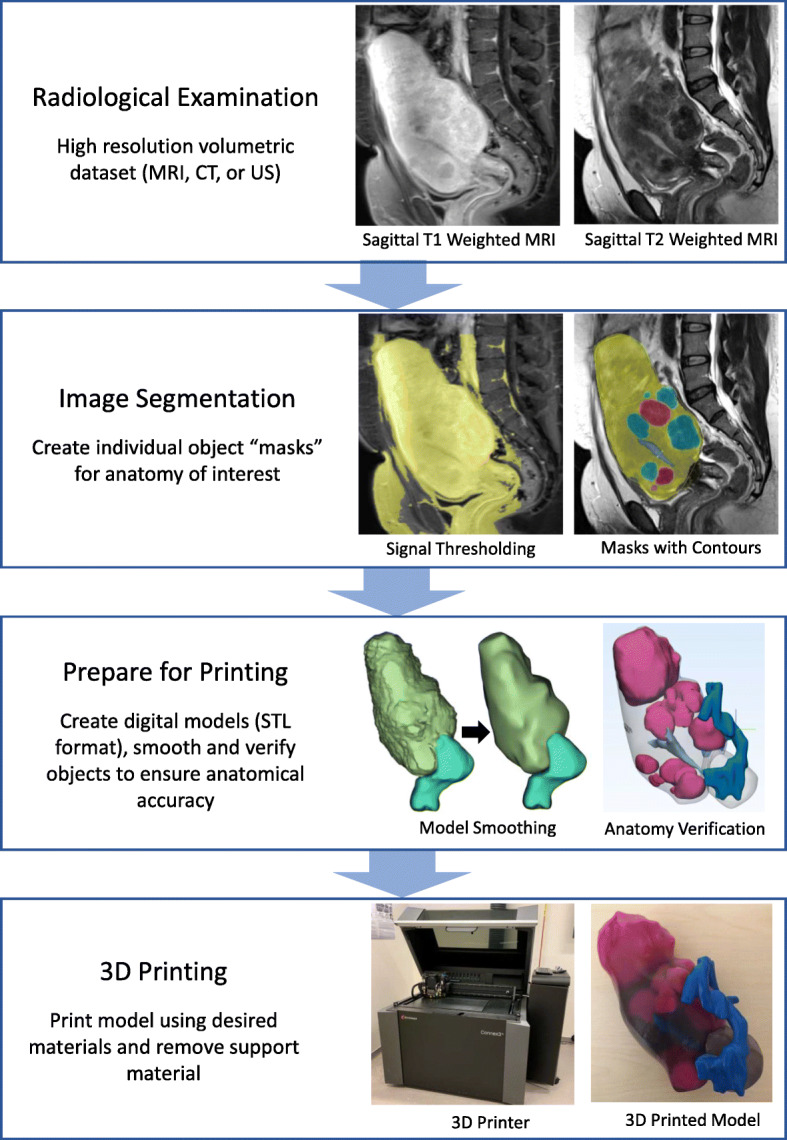
General overview of 3D printing workflow with segmentation of MRI using images of a multi-fibroid uterus as an example

Figure 1 provides a typical workflow for creating anatomical models from DICOM files.

## Application of 3D printing in benign gynecology

### Uterine fibroids

Uterine fibroids are common gynecological tumors affecting up to 80% of women by age 50 worldwide [25]. Although benign, nearly half of these women are symptomatic and experience a significant impact on their quality of life (i.e. heavy menstrual bleeding, dysmenorrhea, chronic pelvic pain, obstructions of adjacent organs, bulk symptoms, and infertility) and require intervention [25–30]. For women with uterine fibroids wishing to preserve their uterus and their fertility, best practice guidelines recommend conservative approaches including medical, surgical or interventional. Myomectomy is the surgical approach to removing fibroids [25], however, complications such as blood transfusion, injury to adjacent organs (bladder, bowel), endometrial perforation, and conversion to hysterectomy can occur in 2–35% of cases [31–33] because of the extent/complexity of disease. Consequently, patients can experience persistent symptoms and require additional surgery in 25–50% of cases [25], and 30% of women may still experience infertility after surgical intervention [31].

**Fig. 2:**
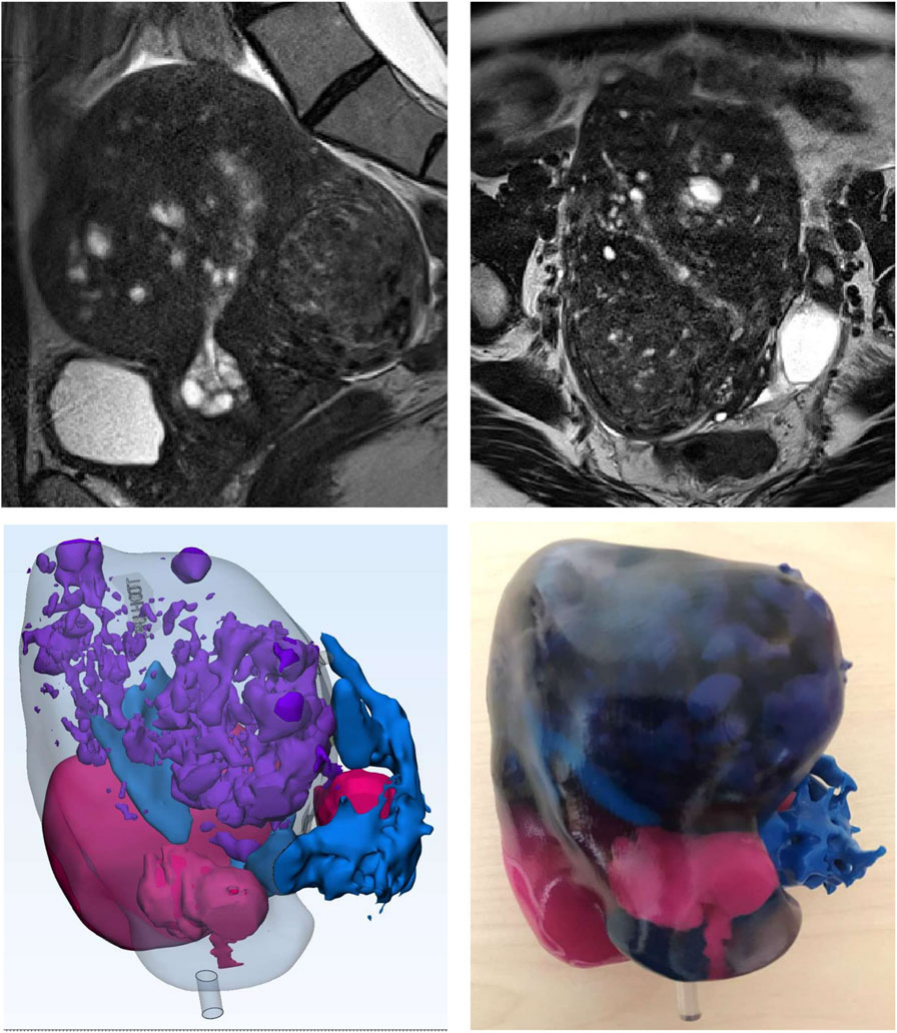
Sagittal (top left) and axial (top right) view of fibroid uterus with adenomyosis depicted on MRI T2 images. MRI images were used to render a 3D digital model (bottom left) and printed as a physical 3D model (bottom right). Adenomyosis was printed in purple, fibroids in magenta, endometrium and external vasculature in blue, and non-neoplastic myometrium and cervical tissue in clear material

**Fig. 3:**
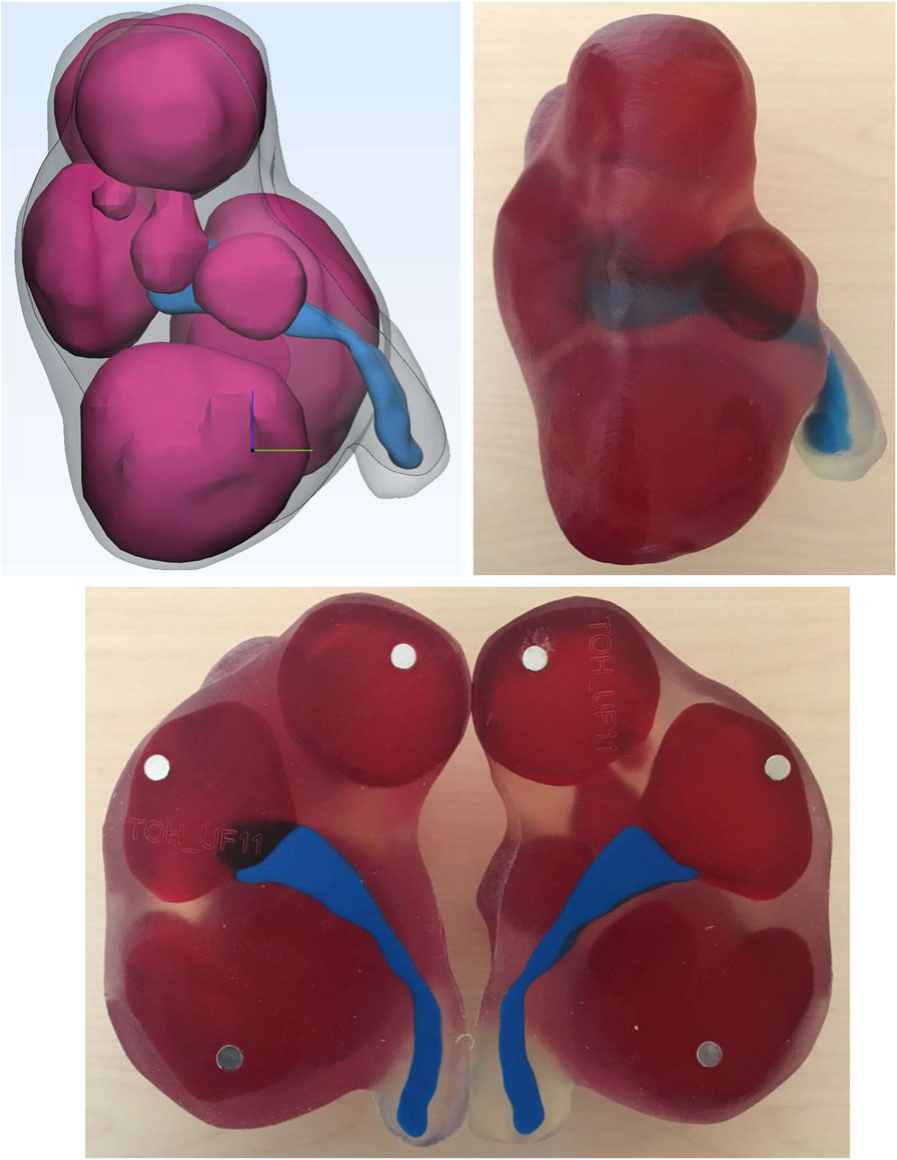
Sagittal plane views of 3D digital model (top left) and 3D printed model (top right). The model was printed in two pieces (bottom), with the mid-sagittal plane as the dissecting line. This was done to improve visualisation of deep fibroids and their position relative to the endometrium. Fibroids were printed translucent red, endometrium in opaque blue, and non-neoplastic myometrium and cervical tissue in clear material. Magnets (grey dots) are used to hold the two pieces together

3D printing is a novel approach for surgical planning of myomectomy or hysterectomy in uterine fibroids as a complementary tool beyond classic 2D imaging modalities. The application of patient-specific 3D printed anatomical models for pre-surgical planning of complex myomectomies has been previously described by our group [34, 35]. Figures 1, 2 and 3 provide examples of our 3D uterine models depicting multiple fibroids. Figure 2 also shows concurrent presence of adenomyosis printed in purple. In our experience, 3D printed models helped surgeons to assess the relationship of the uterine fibroids with surrounding anatomical structures, especially the endometrium and surrounding myometrium. This additional knowledge optimized their excisional course which in turn, minimized allotted surgical time and their perception of estimated blood loss, as well as helped to preserve integrity of the endometrial lining.

Our positive experience of using 3D models for pre-surgical planning and intra-operative reference aligns with previous reports by Aluwee and colleagues [7, 36], who used 3D rendered images and models from MRI for pre-surgical planning. In 10 myomectomy cases, the use of 3D rendered images significantly reduced the surgeon’s time to complete a pre-surgical plan and increased the surgeon’s accuracy of the pre-operative assessment of the disease complexity [7]. In cases of endometrial cancer requiring hysterectomy, surgeons report a positive experience using to models for pre-surgical planning as well as patient education [36]. Similarly, Mackey et al. implemented a patient-specific uterine model to identify the best location for incision during a caesarean delivery complicated by multiple fibroids [37].

### Endometriosis

Endometriosis is a common gynecological disease which affects approximately 15% of females of reproductive age [38], causing symptoms such as, dysmenorrhea, dysuria, dyschezia, dyspareunia, chronic pelvic pain and infertility, which can have a significant impact on their quality of life [30], and a significant economic burden on our healthcare system [39, 40].

Surgery is a common approach for the diagnosis and treatment of endometriosis. Although best practice guidelines recommend a complete surgical excision of all endometriotic lesions in a single surgery, if possible [41, 42], many endometriosis surgeries are incomplete or result in surgical complications because a surgeon may encounter more complex disease than expected. Incomplete and inadequate surgeries lead to persistent symptoms, repeat referral and/or repeat surgery, with more than 60% of patients undergoing multiple operations in an attempt to alleviate the symptoms of pain [43]. Furthermore, laparoscopic excision of deep endometriosis is known to be challenging, even in the hands of highly skilled and well-trained surgeons, as it is both technically demanding and long, with major and minor complications occurring in up to 10% of cases [44, 45].

A thorough understanding of patient-specific anatomy and the extent of the disease is required for developing an appropriate surgical plan. For example, endometriosis affecting the bowel can be surgically managed by shaving, discoid resection, or segmental resection. The lesion(s) size, depth and location (i.e. distance from anal verge) will determine the approach required and if other surgical services need to be consulted [46–48]. However, much of this information is not fully appreciated by the surgeon until direct visualization.

Patient specific anatomical 3D printed models have the potential to improve a gynecologic surgeon’s ability to prepare for highly complex surgical cases involving severe endometriosis. They may allow surgeons to better visualize patient-specific anatomy, the location and extent of endometriotic nodules, suspicion for intra-operative complications, and to optimize intra-operative performance, helping to maximize disease excision while minimizing surgical complications.

To our knowledge, only one other group has created a 3D model of endometriosis. Ajao et al. [49] retrospectively printed a patient-specific anatomical model of a rectovaginal endometriotic nodule from MRI images for a patient with history of endometriosis and persistent pain. Ajao et al. report that the model accurately demonstrated the location and structural relationship of the endometriotic nodule to surrounding structures [49].

**Fig. 4:**
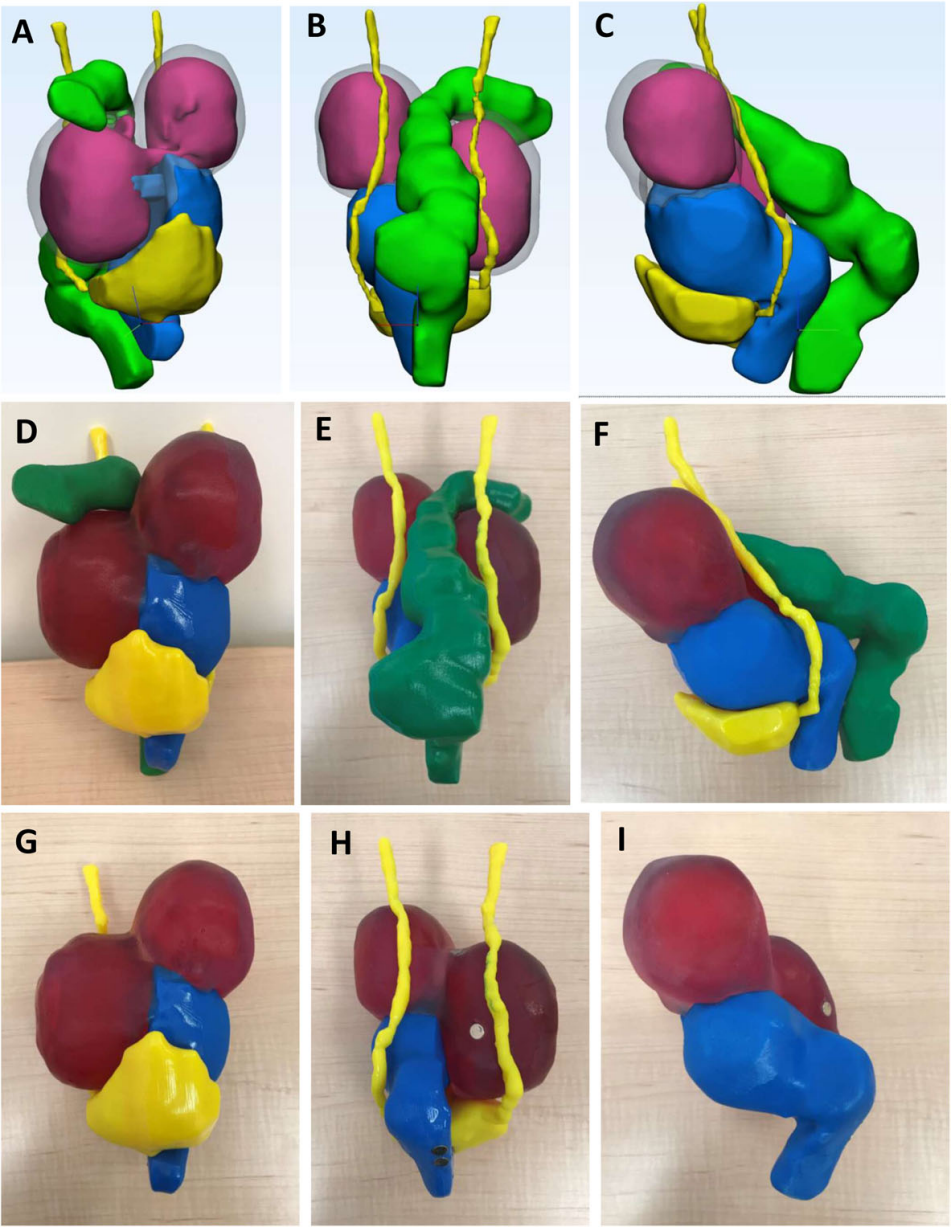
3D digital model for case of deep endometriosis with **a** anterior, **b** posterior and **c** sagittal views. 3D printed model with **d** anterior, **e** posterior and **f** sagittal views. Bladder and ureters were printed in yellow; uterus cervix and vagina printed in blue; bowel and rectum printed in green; endometriomas printed in magenta and ovarian tissue printed in clear material. The model was printed so the bowel and the bladder + ureters could be removed. **g** and **h** show anterior and posterior views without the bowel; **i** is a sagittal view of just the uterus + cervix + vagina and endometriomas

**Fig. 5:**
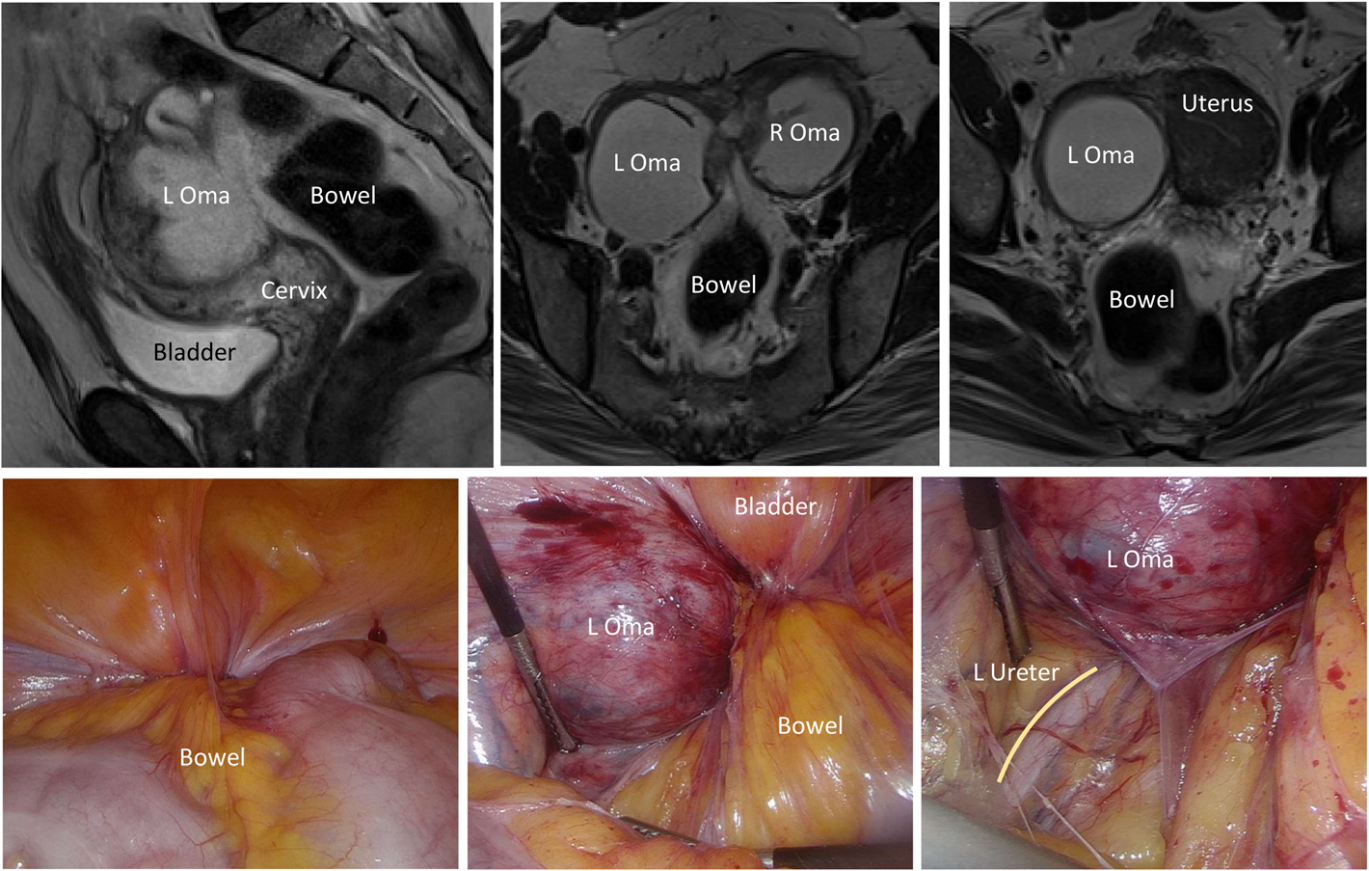
Clinical photos correlating to model in Fig. 4. Sagittal view (top left) and axial views (top middle and top right) of MRI T2 images depicting bilateral endometriomas, distorted bladder, and retroflexion of the uterus’ fundal region. Surgical presentation with no pelvic anatomy seen on initial inspection due to severity of adhesions of the bowel and bladder (bottom left), the bowel adhering to the left endometrioma and bladder (bottom middle), and the close proximity of the left ureter to the left endometrioma (bottom right)

Our group recently created a 3D model for complex endometriosis (Fig. 4). Here we show a case of bilateral endometriomas tethered to the posterior uterus with “kissing ovaries” morphology. The bowel is displaced anteriorly, and the top of the bladder is distorted proximally. Pre-operative medical imaging indicated the bilateral endometriomas, and deep endometriosis between the rectum, uterus, and right ovary causing an obliterated cul-de-sac and bowel tethering to the uterosacral region (Fig. 5). The 3D model correlated with surgical presentation whereby severe adhesions of the bowel and bladder covered the uterine and ovarian structures so pelvic anatomy could not be visualized on initial inspection (Fig. 5). Of note was the close proximity of the left ureter to the left endometrioma reflected in both the model and during the surgery. Our experience with 3D models for cases of deep endometriosis supports observations by Ajao et al. [49]. We believe that 3D models for surgical cases of deep endometriosis can help optimize a surgeon’s pre-surgical plan improving their ability to visualize complex anatomy.

## Barriers to clinical uptake

Two major barriers for the incorporation of 3D models into clinical practice and standard of care are cost and time needed to create the models. Considering material alone, our gynecological models cost between $500.00 and $1700.00 CAD to create at 100% scale, with fibroid models being the most expensive due to enlarged uteri. Our models are comparable in cost to models of the heart with congenital abnormalities [50] and aortic aneurysms [51]. As technology evolves, the cost of 3D printers, supporting software and materials are expected to decrease. Based on our experience, cost-saving considerations can include printing models to 50% scale and printing structures of interest in opaque material (i.e. fibroids) with sparse infill parameters (i.e. internal scaffold).

The average time required to segment our models was approximately 4–6 h and 22 (range: 12–40) h to print. This is comparable to other reports of 3D printing in gynecology [36, 49]. A major limitation contributing to large processing times is the inability to automatically segment gynecological anatomy. Automatic thresholding algorithms are good for segmenting structures with distinct contrast values such as bones and air cavities; though, for soft tissues, available automatic and semi-automatic algorithms are subpar at isolating the various tissue types from the surrounding structures. This is because other tissues within the capture volume have similar signal intensities to gynecological anatomy. An example of this limitation is shown in Fig. 1, left image of second box. Here we tried to isolate the uterine tissue using automatic signal thresholding but surrounding fat, bowel, fascia etc. are also included. Furthermore, differentiating uterine pathologies can be challenging since lesions can present on a spectrum of hypoechoic to hyperechoic relative to the surrounding myometrium [52, 53]. As such, manual corrections are needed to ensure segmentation accuracy which increases processing times and indirectly inflates the cost of 3D gynecological models. In light of this major limitation, recent works have shown promise using deep learning and machine-learning techniques for multi-organ medical image segmentation and medical diagnosis [54, 55]. However, there is a need for high-quality datasets to optimize these algorithms for future application in a clinical setting.

Despite these current limitations, a recent cost-savings analysis by Ballard et al. [56] demonstrated a 60 min mean decrease in surgical time when 3D printed models were used for pre-operative planning or intra-operative surgical guides in orthopaedics or maxillofacial surgery. This decrease in operating time translated to a mean savings of $3720 USD per case. The clinical benefits of reduced operating time and surgical accuracy in a systematic review by Diment et al. [22]. As such, we believe the overall cost of labour, equipment and materials may be balanced by the clinical benefits and reduced burden on the system. Future work on the benefits of 3D printed models in gynecologic surgery should include a cost-effectiveness analysis to demonstrate potential financial benefit of using 3D printed models for reducing operating times and improving patient outcomes on our healthcare system.

## Future directions of 3D printing in gynecology

We see 3D printed models as a novel tool for the preparation and planning of complex surgical procedures, hence having utility which may extend to multiple obstetrical and gynecologic surgeries. For example, several studies have demonstrated the benefit of a multidisciplinary approach involving extensive surgical planning for the optimization of surgical outcomes in cases of placenta accreta spectrum [57, 58]. As such, 3D printed models, which has the potential to improve visualization of maternal, placental and fetal anatomy, may assist in the development of the most appropriate multidisciplinary surgical team, who together can establish a surgical approach which will minimize both maternal and neonatal morbidity and mortality. Additionally, we foresee uses of 3D printing in surgical planning for urogynecology procedures to maximize efficacy and reduce complications of surgeries aimed at treating pelvic floor disorders, and for extensive gynecologic oncology surgeries.

Further applications of 3D printed models in gynecology includes patient education. It is crucial that patients feel a strong understanding of their pathology and plan of care. However, it can be difficult for patients to comprehend complex medical terminology, and 2D radiologic images, which may be provided by their surgeon when they are being counselled in preparation for gynecologic surgery. Aluwee and colleagues [36] created 3D models in preparation for hysterectomy in five patients diagnosed with uterine endometrial cancer and through a questionnaire-based study they showed utility of 3D printed models for both patient education and surgical planning. They reported that patients were satisfied with the uterine 3D models for their understanding of the disease, surgical procedures, and risk of complications. In addition, the surgeons described a favourable experience, reporting that the models facilitated preoperative explanation of surgery to patients and helped obtain information on the positional relationship between the uterus and the tumor [36].

Further, 3D printed models may also benefit surgical trainees, including medical students, residents, and fellows. Within the operating room, surgeons must balance their time spent teaching learners with optimizing patient care and achieving surgical efficiency. Hence, medical educators are constantly in search of strategies and tools to help with teaching surgical trainees. 3D images and models have been used for helping trainees to learn and understand patient specific anatomy for their own surgical planning purposes [59–62]. Additionally, using tissue similar materials, 3D models may be used in simulation-based training, to allow gynecologic procedures to be taught, learned, and practiced outside of the operating room setting.

## Conclusion

To minimize the risk of surgical complications and offer the best health outcomes to patients, successful gynecologic surgery requires a thorough understanding of the patient’s anatomy and burden of disease. A surgeon’s ability to develop a well-thought-out surgical plan is essential to optimizing their intra-operative performance. The adage “most surgery is done in your head” is the motivation behind developing a 3D tool, which has the potential to help the surgeon to mentally rehearse their surgical approach, the planes of dissection, maneuverability, and visibility before making an incision.

We have outlined the utility of 3D printing for surgical planning in gynecology, through two specific examples, uterine fibroids and endometriosis. We also provided a 3D printing protocol to encourage ongoing research and use of 3D printing for this purpose. Further, we have described multiple additional applications of 3D printing in gynecology, outlining the potential unique multi-faceted role 3D printing may have in the specialty in the future.

## Data Availability

Not applicable.
